# Health related quality of life in patients with actinic keratosis – an observational study of patients treated in dermatology specialist care in Denmark

**DOI:** 10.1186/s12955-015-0295-4

**Published:** 2015-07-29

**Authors:** Gunnel Ragnarson Tennvall, J.M. Norlin, I. Malmberg, A.M. Erlendsson, M. Hædersdal

**Affiliations:** IHE, The Swedish Institute for Health Economics, P.O. Box 2127, SE-220 02 Lund, Sweden; LEO Pharma AB, Malmö, Sweden; Department of Dermatology, Bispebjerg Hospital, University of Copenhagen, Copenhagen, Denmark

**Keywords:** Actinic keratosis, Health related quality of life, EQ-5D, DLQI, AKQoL

## Abstract

**Background:**

Actinic keratosis (AK) is a common skin condition that may progress to non-melanoma skin cancer (NMSC). The disease may influence Health Related Quality of Life (HRQoL), but studies of HRQoL in patients with AK are limited.

The purpose of the study was to analyze HRQoL in patients with different severity levels of AK treated in dermatology specialist care using generic and disease-specific HRQoL instruments and to analyze their relationship.

**Methods:**

AK patients who visited dermatological clinics in Denmark were included in an observational, cross-sectional, study in a multi-center setting. Dermatologists assessed AK severity and patients completed: Actinic Keratosis Quality of Life Questionnaire (AKQoL), Dermatology Life Quality Index (DLQI), and EQ-5D-5 L including EQ-VAS. Differences between categorical subgroups were tested with Wilcoxon rank-sum test. The relationship between instruments was analyzed with the Spearman correlation test.

**Results:**

A total of 312 patients were included in the analyses. Patients reported impairment in the disease specific HRQoL instrument AKQoL (mean AKQoL 6.7, DLQI 2, EQ-5D-5 L 0.88, and EQ-VAS 79). HRQoL was least affected in patients with mild actinic disease, whereas patients with severe actinic damage suffered from further impaired HRQoL (mean AKQoL 10.1 and DLQI 4.6). Correlations between DLQI and AKQoL were moderate, whereas the correlations between DLQI and EQ-5D-5 L and between AKQoL and EQ-5D-5 L were weak.

**Conclusions:**

Patients with severe actinic damage showed more impairment in HRQoL than those with mild disease. Correlations between instruments suggest that they are complementary as they measure different aspects of HRQoL and are used for different purposes.

## Background

Actinic keratosis (AK) is a common skin condition associated with cumulative sun exposure. AK lesions possess the risk of progressing to squamous cell carcinoma (SCC) [1–3], which is a common form of non-melanoma skin cancer (NMSC). Whereas AK lesions may regress spontaneously [4], persons who previously suffered from AK have an increased risk of developing new lesions [5]. The prevalence of AK varies considerably and has been estimated to between 1.4 and 25 % of the population [5–9]. In elderly people, in countries with higher ultra violet radiation, and in patients receiving immunosuppressive treatments, such as organ transplant recipients, the prevalence is higher [10].

AKs typically present as red, scaly lesions on visible, sun-exposed skin areas, such as face scalp and dorsal hands, thus often causing cosmetic discomfort. In addition, AK-lesions may itch, bleed, and adhere to clothing and due to its pre-malignant nature patients may fear the risk of developing skin cancer. The presence of AK-lesions may thus influence affected persons’ well-being because of cosmetic reasons, locally hampering symptoms, and also due to fear of developing skin cancer. Studies including patient reported outcome measures and health related quality of life (HRQoL) is increasingly used in clinical practice and in clinical trials in dermatology [11]. Information about HRQoL is requested both by clinicians and reimbursement agencies [12, 13]. Information is limited on the potential impact of AKs, its accompanying symptoms and treatments, on patients’ HRQoL [14–17].

There are several instruments available to investigate HRQoL in dermatology. Generic HRQoL instruments are used for a variety of diseases, which makes it possible to compare the burden of different medical conditions. The EuroQoL five-dimensional questionnaire (EQ-5D) is a generic preference-based instrument, which can be used to calculate quality-adjusted life years (QALYs). QALYs are important in health-economic evaluations and requested by reimbursement authorities [18]. It is recommended to use both generic and disease specific instruments in studies to capture different aspects of HRQoL [19–21]. The EuroQoL Visual Analogue Scale (EQ-VAS) is part of the EQ-5D questionnaire and it measures patient’s overall self-rated health status.

Dermatology-specific or disease-specific instruments include aspects of the HRQoL that may not be captured by a generic instrument. Disease-specific instruments are more responsive to disease activity and treatment outcome and are therefore often used to reflect the patient perspective in clinical trials and observational research.

The Dermatology Life Quality Index (DLQI) is widely used in dermatology, both in clinical practice and in research [22, 23] and it has recently been used in patients with AK [24]. The questions in the DLQI focus on physical limitations, rather than psychological impact of skin diseases.

Recently, the Actinic Keratosis Quality of Life Questionnaire (AKQoL) was developed [14]. This questionnaire reflects how sun-damaged skin affects HRQoL and has the primary focus on psychological aspects.

The objective of the study was to analyze HRQoL in patients with different severity levels of AK treated in dermatology specialist care using generic and disease-specific HRQoL instruments and to analyze their relationship.

## Methods

### Study set-up

This is an observational cross-sectional multi-center study with focus on clinical patient characteristics and patient reported outcome measures. Data was collected at three university hospital clinics and seven private dermatology clinics in three geographic regions (Zealand, Funen and Jutland) in Denmark. AK patients ≥18 years who visited the clinics during one specific week in May/June in 2012 were included.

### Ethics, consent and permissions

The study was carried out after obtaining approval from The Danish Data Protection Agency. All persons gave their informed consent prior to inclusion in the study.

### Data collection

Patients were asked to complete three separate HRQoL questionnaires i) AKQoL, ii) DLQI and iii) EQ-5D with 5 response levels (EQ-5D-5 L), including EQ-VAS.

Information was collected about educational level, employment, civil status and the following chronic co-morbidities; asthma or chronic obstructive pulmonary disease; heart or vascular disease; diabetes; gastro-intestinal disease; cancer; joint disease (e.g. osteoarthritis, rheumatoid arthritis); physical disability or other chronic disease.

In order to characterize the severity of actinic damage, physicians collected information about previous and current AK lesions. Presence of AK was evaluated separately in nine anatomical regions (scalp, ears, face, chest, trunk, arm, hand, leg and foot), and for each AK-affected region, the following was registered: lesion count, lesion thickness, presence of field-cancerization, clinical suspicion of NMSC and selected treatment(s). Lesion count was estimated and categorized as 1, 2–4, 5–20, or >20 lesions per area. AK-thickness was graded from I-III according to Olsen et al. [25]. Field cancerization was classified as mild, moderate or severe, based on the clinical presentation of surrounding skin and included mottled erythema and pigmentation, telangiectasia, sallowness, laxity, and dry skin texture, without fulfilling the definition of AK. In the present study, ‘Severe Actinic Damage’ was defined as an anatomical area with >5 lesions, dominated by grade II-III AKs with moderate to severe field cancerization. Consequently, ‘Mild actinic damage’ was defined as an anatomical area with a single grade I AK without field cancerization. Treatment and disease characteristics were the focus in a separate publication [26].

### Patient reported outcome measures of health related quality of life

AKQoL is an AK specific HRQoL questionnaire including 9 questions with one single global item and three subscales/domains: function, emotions and control [14]. The questionnaire is reflecting personal daily life, personal view of quality of life, social life, emotional life and control of life [14]. Each question is scored on a 4-point scale: A lot/all the time (scores 3), Quite a lot/often (scores 2), Some/sometimes (scores 1), Rarely/not at all (scores 0). In case only one question is incomplete, the missing value is designated a score of zero and the patient is included in the analysis [14]. A total score ranging from zero to a maximum of 27 is calculated by summing the score of each question. The higher the score, the more severe is the HRQoL impairment. The AKQoL includes the three domains Function, Emotions, and Control, which are summarized into one single Global item.

DLQI is a dermatology-specific instrument that relates to how the skin disease has affected the life of the patient over the past 7 days [23]. The questionnaire consists of 10 questions in 6 dimensions: 1) Symptoms and feelings, 2) Daily activities, 3) Leisure, 4) Work and school, 5) Personal relationships, and 6) Treatment. Each question has 4 alternative answers: “not at all”, “a little”, “a lot” and “very much”, scored 0, 1, 2 and 3, respectively. The overall score aggregates the score of each question. DLQI ranges from 0 to 30, where a higher score represents more severe HRQoL impairment.

EQ-5D is a generic HRQoL instrument. The questionnaire includes five dimensions, mobility, self-care, usual activities, pain/discomfort, and anxiety/depression [27]. The Danish version of the EQ-5D-5 L was used in the study. The EQ-5D-5 L questionnaire has the same structure as the traditional three level questionnaire (EQ-5D-3 L), but with five response levels: no problems, slight problems, moderate problems, severe problems and unable to/extreme problems. Each combination of responses is associated with utility values or relative weighting, which are often derived from previous population-based studies. Utility values are usually expressed on a scale ranging between 0 and 1, where a higher value represents better HRQoL but it may result in a value lower than zero indicating a health state considered worse than dead. The utility values make it possible to calculate QALYs, which are essential in health economic evaluations of health care interventions [18]. As utility values for the EQ-5D-5 L were not yet available at the time of analysis, a Danish value set, developed by the EuroQol group, was used to transform values from the EQ-5D-3 L to the EQ-5D-5 L version [28].

In the EQ-VAS part, patients were asked to indicate their present health state on a vertical scale, numbered from 0 to 100, where 100 is “the best imaginable health state” and zero “the worst imaginable health state”.

### Data analysis and statistical methods

Data analyses of background information and subgroup analyses of HRQoL outcomes were performed using descriptive statistical methods, using mean values, standard deviations (SD) and proportions.

The AKQoL, DLQI, and EQ-5D-5 L values were analyzed overall and for subgroups of patients depending on sex, age-groups (<60, 60–69, 70–79 and ≥80), current AK at study visit, clinically suspected NMSC, lesion/s in the face, current comorbidities, current AK treatment, immunosuppressive treatment, and previous SCC or not. In addition, analyses of HRQoL in subgroups of patients with severe actinic damage or not was performed.

Statistically significant differences between categorical subgroups were tested with Wilcoxon rank-sum test (Mann–Whitney U test). For age groups a Kruskal-Wallis test was used, which is an extension of the Wilcoxon rank-sum test to several groups. Non-parametric tests were chosen as the HRQoL outcomes were not normally distributed.

Correlations between instruments were tested with the Spearman correlation test to investigate whether there was a relationship between them. The Spearman correlation test gives an absolute value between 0 and 1, where 0 indicates no correlation and 1 indicates perfect correlation. The relationship could be positive or negative. A high EQ-5D-5 L or EQ-VAS value means good HRQoL, whereas a high score on DLQI or AKQoL indicates low HRQoL. Correlations between EQ-5D-5 L or EQ-VAS and DLQI or AKQoL therefore have negative values.

A multiple ordinary least squares (OLS) regression analysis with EQ-5D-5 L as the dependent or outcome variable was performed in order to investigate how EQ-5D-5 L was affected by age, sex, comorbidities, severe actinic damage, and clinically suspected NMSC. Reference group was man, no comorbidities, no severe actinic damage, and no NMSC. Only patients with current AK lesions were included in regression analysis.

A p-value lower than 0.05 was used as significance level in all statistical analyses. Statistical analyses were performed using Stata Statistical Software: Release 11.1. College Station, Texas, USA.

## Results

### Patient characteristics and background

Patient characteristics are presented in Table 1. A total of 312 patients were enrolled in the study. The majority of patients, 89 %, (*n* = 277), had current AK lesions at the study visit. The remaining 35 patients had attended control visits for previous AK. Of all patients, 80 % had a pre-history of AKs and 42 % suffered from recurrence of a specific AK lesion.

**Table 1:** Patient characteristics

	*N*	Percent
	312	100
Women	160	51
Age, mean (SD)^a^	71 (11.0)	
Immunosuppressive treatment	28	9
Current AK lesion/s	277	89
Previously known AK	251	80
Previous AK treatment	245	78
Recurrent AK	130	42
Previous SCC	58	19
Previous BCC	135	43

*SCC* Squamous Cell Carcinoma, *BCC* Basal Cell Carcinoma

^a^One missing value

Sixty-seven percent were retired, 16 % were working, 6 % reported other activities and 11 % did not report employment status. Comorbidities were reported in 66 % of patients. The most common comorbidity was heart or vascular disease (26 %), followed by joint disease (22 %) and cancer (21 %). Nine percent were treated with immunosuppressive treatments.

A summary of the results from the different HRQoL instruments is shown in Table 2.

**Table 2:** Overall mean (SD) AKQoL, DLQI, EQ-5D-5 L, and EQ-VAS values and in subgroups of patients

		AK-QoL				DLQI				EQ-5D-5 L				EQ-VAS			
		*n*	Mean	SD	*p*-value	*n*	Mean	SD	*p*-value	*n*	Mean	SD	*p*-value	*n*	Mean	SD	*p*-value
Total population		286^a^	6.7	4.8		290^b^	1.99	2.71		276^c^	0.884	0.140		284	79.3	18.9	
Sex	Men	137	5.3	3.7	<0.001	138	1.73	2.46	0.121	132	0.901	0.127	0.060	136	81.3	17.9	0.086
	Women	149	7.9	5.3		152	2.23	2.91		144	0.868	0.150		148	77.6	19.7	
Age groups^d^	<60	38	8.1	5.5	0.004	39	2.31	2.40	0.657	37	0.903	0.126	0.262	38	77.6	22.9	0.070
	60-69	103	6.6	4.7		102	2.27	3.36		98	0.895	0.134		102	81.9	16.7	
	70-79	86	7.3	4.7		87	1.75	2.38		84	0.888	0.139		85	81.1	18.1	
	≥80	58	5.0	4.0		61	1.70	2.04		56	0.850	0.157		58	73.7	20.2	
Current AK	Yes	252	6.5	4.9	0.018	257	2.13	2.83	0.009	244	0.881	0.140	0.229	252	79.0	19.3	0.553
	No	34	7.8	3.7		33	0.91	1.04		32	0.907	0.141		32	82.2	15.5	
Severe actinic damage^e^	Yes	27	10.07	5.6	<0.001	27	4.59	4.07	<0.001	26	0.844	0.139	0.068	27	70.2	22.3	0.012
	No	259	6.30	4.5		263	1.73	2.38		250	0.888	0.147		257	80.3	18.3	
Suspected NMSC^f^	Yes	38	6.0	5.2	0.259	39	2.64	4.07	0.950	37	0.856	0.160	0.343	39	71.8	22.6	0.030
	No	214	6.6	4.8		218	2.04	2.54		207	0.886	0.136		213	80.3	18.4	
Locations^f^	Face	178	6.5	4.7	0.640	182	2.11	2.68	0.531	170	0.884	0.133	0.784	179	79.4	19.0	0.671
	Non-facial	74	6.5	5.2		75	2.19	3.17		74	0.873	0.156		73	78.0	20.1	
Comorbidities^f^	Yes	175	6.7	4.9	0.357	178	2.38	3.19	0.200	170	0.860	0.151	<0.001	175	74.9	20.1	<0.001
	No	77	6.1	4.7		79	1.57	1.64		74	0.930	0.097		77	88.1	13.4	
Current AK treatment	Yes	125	7.0	5.6	0.402	127	2.43	3.38	0.186	120	0.900	0.130	0.221	123	78.2	20.3	0.609
	No	74	5.8	3.7		74	1.73	2.14		72	0.870	0.150		73	81.2	16.5	
Immunosuppressive treatment^f^	Yes	25	7.2	6.0	0.869	25	4.04	4.46	0.023	23	0.876	0.143	0.900	24	70.6	25.1	0.061
	No	227	6.4	4.7		232	1.93	2.52		221	0.881	0.140		226	80.1	18.3	
Previous SCC	Yes	53	8.0	6.6	0.410	54	3.4	4.4	0.016	51	0.849	0.153	0.038	52	70.8	21.4	<0.001
	No	233	6.4	4.2		236	1.7	2.0		225	0.892	0.136		213	81.3	17.9	

^a^26 incomplete questionnaires, ^b^22 incomplete questionnaires, ^c^36 incomplete questionnaires, ^d^One missing value, ^e^Severe actinic damage defined as patients with multiple lesions, dominated by grade II-III AKs with moderate to severe field cancerization. ^f^Analysis of “Suspected NMSC”, “Locations”, “Comorbidities” and “Immunosuppressive treatment” only included patients who had current AK lesion/s at the study visit and who had answered the respective questionnaire

### The actinic keratosis quality of life questionnaire

The mean AKQoL score for the 286 patients who completed the questionnaire was 6.7 (scale: 0–27). Patients with severe actinic damage had worse HRQoL (10.07) than patients with no severe actinic damage (6.3) (*p* < 0.001).Women reported higher AKQoL scores (7.9), i.e. worse HRQoL, than men (5.3) (*p* < 0.001). The scores also differed between age groups with patients younger than 60 years of age reporting worse HRQoL (8.1) compared to older patients (5.0-7.3) (*p* = 0.004). Patients with current AK reported better HRQoL (6.5) than patients without current AK (7.8) (*p* = 0.018).

**Fig. 1:**
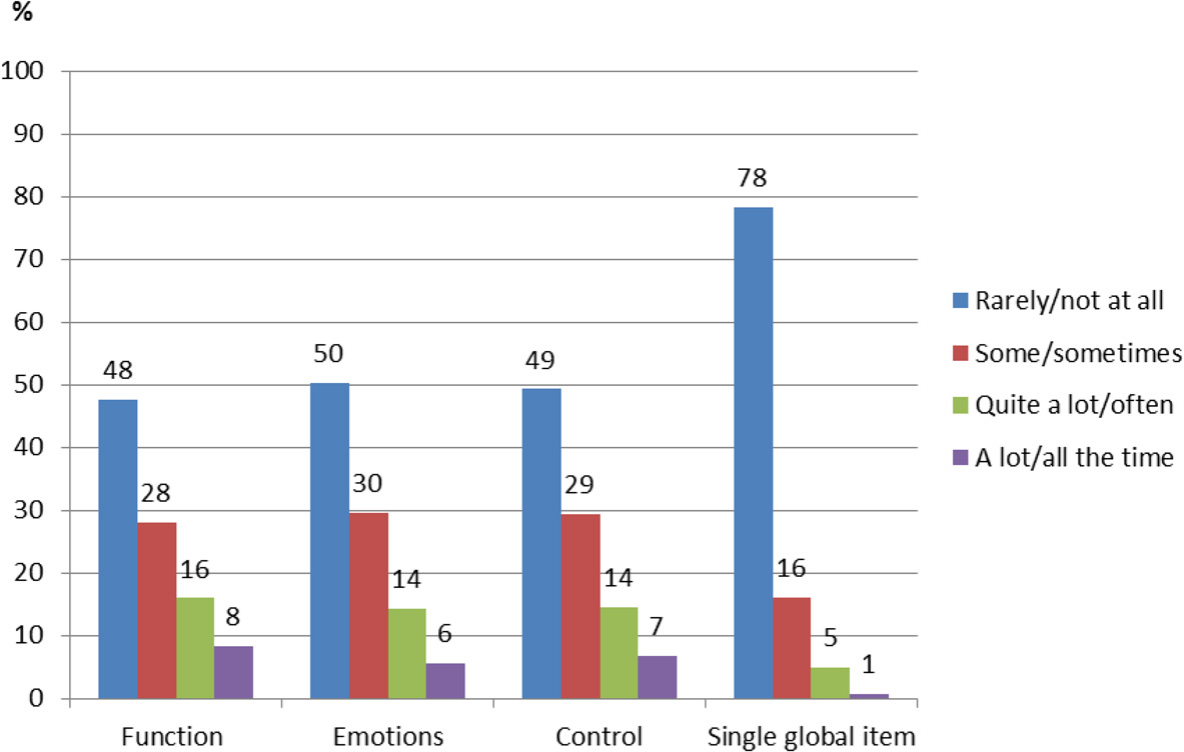
The percentage of response levels in each domain of the AKQoL score. 26 questionnaires had more than one incomplete question. Six patients, out of the 286, had one missing value only in one of the domains and were included in the analysis

The distribution between the different response levels was similar in the different domains function, emotions and control (Fig. 1). About 50 % reported problems in each domain and in the single global item, 22 % reported impairment in HRQoL.

### Dermatology life quality index

**Fig. 2:**
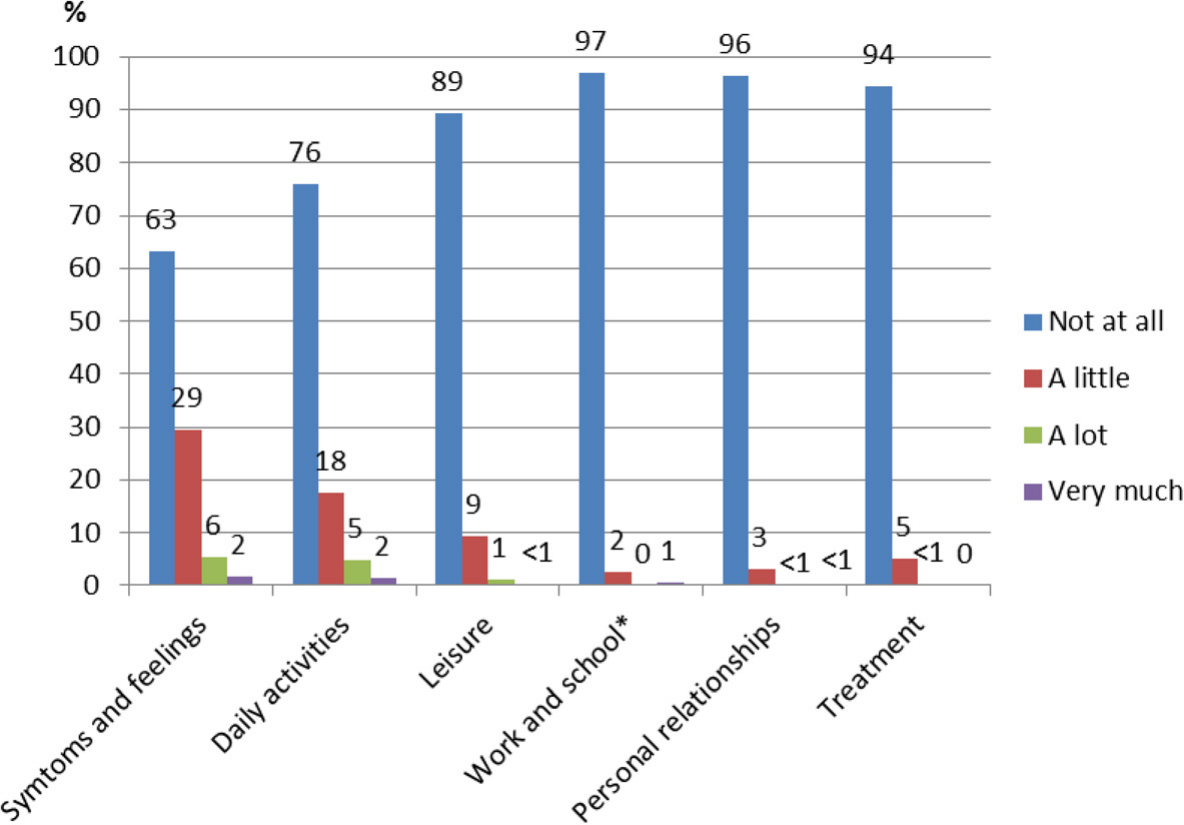
The percentage of response levels in each dimension of the DLQI score. n=290 22 incomplete questionnaires

The mean value for the 290 patients who completed the DLQI questionnaire was 1.99 (scale: 0–30). Patients with severe actinic damage had higher DLQI scores (4.6), i.e. worse HRQoL, than patients with milder AK (1.7) (*p* < 0.001). Likewise, patients with current AK had higher DLQI (2.1) than patients with no current AK lesions (0.9) (*p* = 0.009). Patients with previous SCC had higher DLQI scores (3.4) than those without previous SCC (1.7) (*p* = 0.016) and patients treated with immunosuppressive treatments had higher DLQI scores (4) than patients who were not treated with such drugs (1.9) (*p* = 0.023). “Symptoms and feelings” was the DLQI dimension where most patients reported HRQoL impairment (37 %). The second most reported dimension related to the skin disease was “Daily activities” (25 %) (Fig. 2).

### EQ-5D-5 L and EQ-VAS

**Fig. 3:**
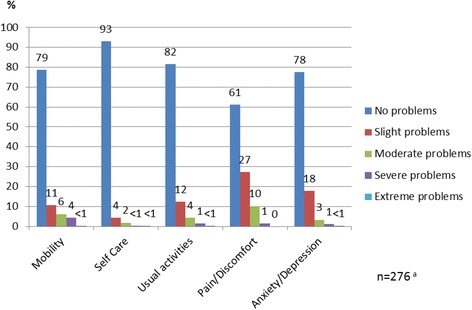
The percentage of response levels in each dimension of EQ-5D-5 L. ^a^ 36 incomplete questionnaires

For the 276 patients who completed the EQ-5D-5 L questionnaire, the mean value was 0.88 (scale: 0–1). Patients with comorbidities reported lower HRQoL (0.86) than those without comorbidities (0.93) (*p* < 0.001). Likewise, patients with previous SCC had lower HRQoL (0.85) than those without previous SCC (0.89) (*p* = 0.038). Patients reported most problems in the Pain/Discomfort dimension (38 %) (Fig. 3).

The overall EQ-VAS value for 284 responding patients was 80. For patients with severe actinic damage the corresponding value vas 70.

### Correlations between instruments and regression analysis with EQ-5D-5 L as outcome

The correlations between the HRQoL instruments are presented in Table 3. The analyses showed statistically significant correlations in all comparisons except between EQ-5D-5 L and AKQoL and between EQ-VAS and AKQoL. The correlation between DLQI and AKQoL was strongest, 0.52 (*p* < 0.001). This indicates that DLQI and AKQoL partly measure the same aspects of HRQoL. The correlation between EQ-5D-5 L and DLQI was weaker, −0.36 (*p* < 0.001) but showing that there is a relationship between a higher EQ-5D-5 L value and a lower DLQI value. A similar correlation pattern, but weaker, was shown between EQ-VAS and DLQI, −0.21 (<0.001).

**Table 3:** Correlations between the HRQoL instruments, EQ-5D-5 L, EQ-VAS, DLQI, and AKQoL, tested with Spearman correlation test

HRQoL instruments	Correlation	*p*-value	*N*
EQ-5D-5 L - DLQI	−0.36	<0.001	273
EQ-5D-5 L - AKQoL	−0.10	0.099	270
EQ-VAS - DLQI	−0.21	<0.001	282
EQ-VAS – AKQoL	−0.01	0.859	278
DLQI - AKQoL	0.52	<0.001	283

The regression analysis with EQ-5D-5 L as the dependent variable showed that HRQoL decreased significantly with increasing age (−0.002 per year; *p* = 0.007), being a woman (−0.039; *p* = 0.026) and comorbidity (−0.063; *p* = 0.001).

## Discussion

This observational, multi-center, cross-sectional study, showed that AK, especially severe actinic damage, has a negative impact on HRQoL, as measured by the AK and dermatology specific HRQoL instruments. This is demonstrated in several of the dimensions or domains in the different instruments and indicate that the disease specific and dermatological instruments capture different aspects of HRQoL.

To our knowledge, this is the first study where dermatologists have assessed the overall severity level of the AK disease in patients, rather than the severity grade in particular lesions only. Furthermore, it is the first study, to assess HRQoL with both generic and disease specific instruments in patients with AK. Results from all instruments showed more impaired HRQoL in patients with severe actinic damage than in those who were defined as having mild disease. The difference was statistically significant for AKQoL and DLQI.

The strength of this study is that patients can be considered representative for a dermatological population as they were included from different dermatology clinics in different parts of Denmark. A weakness of the study is that the sample in some subgroup analyses was limited, and may therefore limit the possibility of statistically significant results.

The only previously published study including the AKQoL questionnaire describes the development and validation of the questionnaire [14]. The mean AKQoL score in that study was 9.5, which is similar to the score of 10.07 reported in the present study for patients with severe actinic damage. The AKQoL questionnaire is focused on the anxiety associated with sun damaged skin and the risk of developing a more serious disease. Contrary to what one would expect, patients with current AK lesions had lower impairment in AKQoL than patients with no current lesions. The reason for this may be that patients who previously have had lesions may still be worried about their skin as the risk of developing a new lesion is high [5]. The AKQol may therefore not be sensitive to change in clinical outcomes. Further evaluation and validation of the AKQoL questionnaire is needed before any recommendations about the usefulness of the instrument can be made.

DLQI is commonly used in dermatology studies [20, 22, 29–35], but we found only two studies including AK patients [24, 36]. In the first study DLQI was 1.95 for AK patients who received PDT with aminolevulinic acid and 1.38 for patients treated with imiquimod [36], in comparison to the mean value 1.99 for all patients and 4.59 for patients with severe actinic damage in the present study. In the second study DLQI varied from 1.6 prior to photodynamic therapy to 7.3 post treatment but was then normalized [24]. DLQI values between 2 and 5 could be interpreted as low effect on patient’s life while values from 6 to 10 could be interpreted as having moderate effect [23]. In our cross-sectional study, there was no statistically significant difference in DLQI among patients who were currently in treatment and patients who had completed the treatment. More research is needed about how different treatment options affect patients HRQoL. Previous studies of patients with NMSC showed mean DLQI values of 2.4 and 4.9 [31, 32]. In observational studies of psoriasis and eczema DLQI ranged from 4.5 to 6.8 [20, 33, 34]. In a study of acne including a young population (mean age 22), the mean DLQI was 9.2 [35]. In comparison with other dermatological diseases such as NMSC, psoriasis and eczema, patients with severe actinic damage thus have similar HRQoL, but better HRQoL than young persons with acne.

EQ-5D has previously been used in a variety of studies of dermatologic conditions [20, 34, 35, 37–42], but we found no study of AK. The burden of disease varies between different skin conditions, with EQ-5D ranging from 0.43 in herpes zoster [40] to 0.84 in acne [35] and 0.85 in the mildest form of atopic dermatitis [39]. For severe actinic damage the mean EQ-5D value of 0.84 is similar to both acne and mild atopic dermatitis. The mean value of 0.88 in the overall patient group is similar to the 0.89 in the general Danish population [43]. A recent literature review of the use of EQ-5D in economic evaluations in dermatology included 20 studies identified between 2003 and 2011. The authors suggested that although the EQ-5D is broad enough to allow comparison between different diseases, it may not be specific enough to capture important aspects of HRQoL in dermatology [44].

One limitation of comparisons across results from different studies is that the patient populations might not be completely comparable regarding characteristics such as age, sex and co-morbidity. In the general population, women tend to report lower HRQoL than men and elderly lower HRQoL than younger age groups [43, 45]. In dermatology-specific HRQoL, however, younger individuals have reported lower HRQoL than older age groups [20]. This tendency can also be observed in our results, especially in the AKQoL instrument where the difference in HRQoL between patients younger than 60 years of age and older patients was statistically significant.

The correlation analyses suggest that the instruments EQ-5D-5 L, DLQI and AKQoL are complementary as they measure different aspects of the HRQoL. The magnitude of the correlation between DLQI and AKQoL, 0.52, can be interpreted as moderate [46], while the correlations between the DLQI and EQ-5D-5 L, −0.36, and between DLQI and EQ-5D-5 L, −0.21, can be interpreted as weak [46]. This is lower than previous findings of correlations −0.51 to −0.55 between EQ-5D-5 L and DLQI in patients with psoriasis [20, 34, 41]. There was no or little correlation between EQ-5D-5 L and AKQoL, which was expected as the EQ-5D captures overall HRQoL, and includes aspects beyond the impact of the skin.

The role of EQ-5D in economic evaluations in dermatology has been questioned as the EQ-5D is broad enough to allow comparison between different diseases, but it may not be specific enough to capture important aspects of HRQoL in dermatology [44]. Therefore, it is important to use both generic, dermatology- and disease specific HRQoL measure in dermatologic conditions such as AK.

The generic EQ-5D is important for health economic evaluations and it is essential as long as it is preferred by reimbursement authorities and policy makers for comparing costs and benefits across medical conditions [12, 13]. Moreover, generic instruments also measure comorbidities that go beyond conditions of the skin.

The DLQI is dermatology specific and is therefore more sensitive to detect changes in HRQoL related to clinical outcomes in dermatological conditions, which could be useful for regulatory authorities and for clinicians to individualize interventions and provide optimal care for patients. Moreover, as it is one of the most commonly used instruments in dermatology, the DLQI is appropriate for comparison with other skin diseases.

Whereas, the DLQI has been criticized of being too focused on physical limitations rather than the psychological impact of the skin [47], the AKQoL is focused on emotions and worries related specifically to sun damaged skin. The AKQoL captures domains of the HRQoL which are of relevance for persons with AK as they may worry about lesions developing to SCC. Furthermore, since the AK population is relatively old, some questions in the DLQI such as how much the skin caused problems in sports, work or school, and sexual relationships might be of less importance for an elderly population.

## Conclusions

The EQ-5D-5 L, DLQI and AKQoL provide complementary information and are all useful, as they capture different aspects of HRQoL. Whereas the EQ-5D is essential for economic evaluations, the DLQI is responsive to change in relation to treatment and the AKQoL captures important features of the HRQoL that are specifically related to sun damaged skin. Future research is needed to further evaluate the responsiveness to change of the DLQI in relation to treatment and the AKQoL needs to be validated in future clinical studies.

The present study has shown that patients with severe actinic damage have impaired HRQoL, while patients with mild disease are less affected. HRQoL in patients with severe AK is similar to HRQoL in patients with psoriasis and eczema.

## Abbreviations

AK: Actinic keratosis
AKQoL: Actinic keratosis quality of life questionnaire
BCC: Basal cell carcinoma
CRF: Case report form
DLQI: Dermatology life quality index
EQ-5D-5 L: EuroQoL five dimensions five levels
EQ-VAS: EuroQoL visual analogue scale
HRQoL: Health related quality of life
NMSC: Non-melanoma skin cancer
OLS: Ordinary least squares
PDT: Photodynamic therapy
QALY: Quality adjusted life years
SCC: Squamous cell carcinoma
SD: Standard deviation
VAS: Visual analogue scale
